# Artificial intelligence-integrated video analysis of vessel area changes and instrument motion for microsurgical skill assessment

**DOI:** 10.1038/s41598-025-13522-1

**Published:** 2025-07-31

**Authors:** Taku Sugiyama, Minghui Tang, Hiroyuki Sugimori, Marin Sakamoto, Miki Fujimura

**Affiliations:** 1 Department of Neurosurgery, Hokkaido University Graduate School of Medicine, North 15 West 7, Kita-ku, Sapporo, 060-8638 Japan; 2 Medical AI Research and Development Center, Hokkaido University Hospital, Sapporo, Japan; 3 Department of Diagnostic Imaging, Hokkaido University Faculty of Medicine and Graduate School of Medicine, Sapporo, Japan; 4 Department of Biomedical Science and Engineering, Faculty of Health Sciences, Hokkaido University, Sapporo, Japan; 5 Graduate School of Health Sciences, Hokkaido University, Sapporo, Japan

**Keywords:** Deep learning, EC-IC bypass, Microsurgical training, Objective surgical skill evaluation, Surgical education, Tissue deformation, Neurosurgery, Software

## Abstract

Mastering microsurgical skills is essential for neurosurgical trainees. Video-based analysis of target tissue changes and surgical instrument motion provides an objective, quantitative method for assessing microsurgical proficiency, potentially enhancing training and patient safety. This study evaluates the effectiveness of an artificial intelligence (AI)-based video analysis model in assessing microsurgical performance and examines the correlation between AI-derived parameters and specific surgical skill components. A dual AI framework was developed, integrating a semantic segmentation model for artificial blood vessel analysis with an instrument tip-tracking algorithm. These models quantified dynamic vessel area fluctuation, tissue deformation error count, instrument path distance, and normalized jerk index during a single-stitch end-to-side anastomosis task performed by 14 surgeons with varying experience levels. The AI-derived parameters were validated against traditional criteria-based rating scales assessing instrument handling, tissue respect, efficiency, suture handling, suturing technique, operation flow, and overall performance. Rating scale scores correlated with microsurgical experience, exhibiting a bimodal distribution that classified performance into good and poor groups. Video-based parameters showed strong correlations with various skill categories. Receiver operating characteristic analysis demonstrated that combining these parameters improved the discrimination of microsurgical performance. The proposed method effectively captures technical microsurgical skills and can assess performance.

**Supplementary Information:**

The online version contains supplementary material available at 10.1038/s41598-025-13522-1.

## Introduction

A surgeon’s technical proficiency is critical and has been linked to postoperative adverse events, morbidity, and mortality^1,2^. In extracranial-intracranial (EC-IC) bypass surgery, the fragility of cerebral arteries necessitates advanced microsurgical skills to achieve complete intimal approximation between the recipient and donor arteries^3–5^. This demanding technique requires precise instrument handling, smooth and efficient movements, and gentle tissue manipulation to prevent vessel wall tears^6,7^. A comprehensive, objective assessment of microsurgical skills provides a reliable means to identify deficiencies in surgical trainees through constructive feedback and validates surgeon proficiency, ultimately enhancing patient safety^8^.

Traditionally, microsurgical skill assessments have relied on subjective evaluations by master surgeons^9^. Although various criteria-based scoring systems have been developed to reduce subjectivity, they require substantial human and time resources, making real-time feedback impractical^10–13^. Quantitative methodologies, such as force and motion sensors affixed to surgical instruments, have been explored^14–16^; however, their reliance on specialized sensors and equipment limits their widespread adoption and raises concerns regarding generalizability and reproducibility^17,18^.

With the increasing availability of surgical video recordings, video analytics is gaining traction for skill assessment across various procedures^19–21^. Artificial intelligence (AI)-driven video analysis is also being increasingly applied in surgical skill evaluation^22,23^. Building on this trend, we previously developed two AI models for assessing microvascular anastomosis performance: one incorporating a semantic segmentation algorithm to evaluate vessel area (VA) changes^24^ and another using an object detection algorithm to analyze surgical instrument-tip motion^25^.

This study examines whether combining these AI models improves the accuracy of surgeon performance assessment and identifies which aspects of microsurgical skills correlate with AI-derived parameters.

## Methods

### Combined AI model

Two AI models were developed as described previously: a semantic segmentation algorithm for the VA and a trajectory tracking algorithm for the instrument tip^24,25^. These models were based on the Residual Network 50 (ResNet-50) and You Only Look Once version 2 (YOLOv2), which were trained on clinical microsurgical videos and microvascular anastomosis practice videos.

ResNet-50 is a 50-layer deep convolutional neural network that utilizes residual learning, enabling the training of very deep architectures, and is widely applied to tasks such as segmentation and classification in medical imaging^26^. YOLOv2 is a real-time, deep learning-based object detection algorithm designed to detect objects quickly and accurately in a single pass. It divides the input image into grids and simultaneously predicts the presence of objects within each grid cell, making it ideal for high-speed, real-time applications such as surveillance systems for detecting people and vehicles, and identifying anatomical structures in medical images^27^. Both models were implemented in MATLAB (MathWorks, Natick, MA, USA). Detailed training procedures for each model can be found in our previous studies^24,25^.

**Fig. 1:**
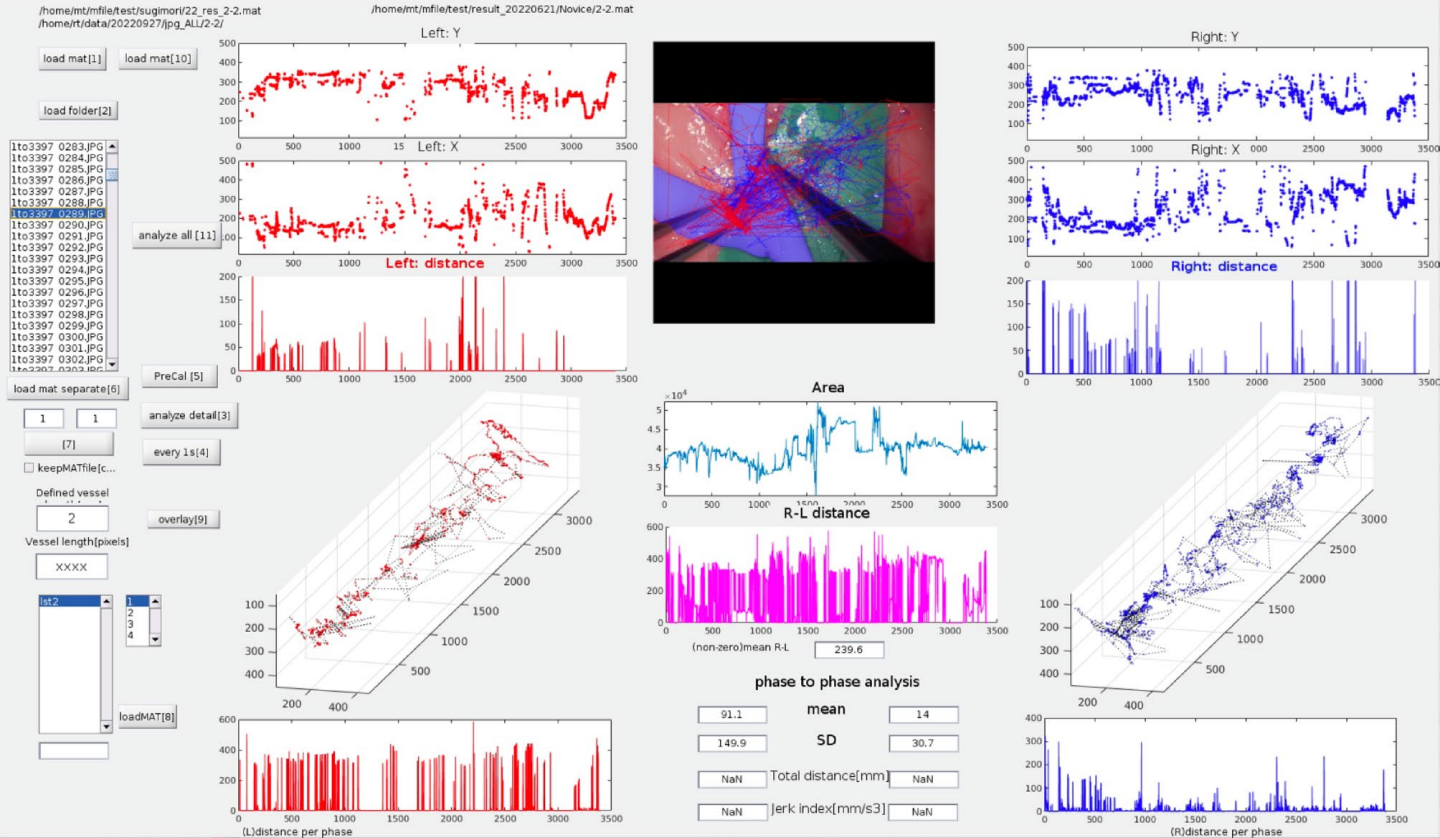
Graphical user interface of the custom-built software, showcasing the artificial intelligence (AI)-powered vessel segmentation and instrument tracking models. The interface provides real-time visualization of vessel area changes and instrument motion for microsurgical performance assessment.

The accuracy of these models was ensured by an Intersection over Union of 0.93 and a mean Dice similarity coefficient of 0.87^24,25^. We integrated both AI models to comprehensively analyze the microsurgical performance, as shown in Fig. 1.

### Participants

This study adhered to the SQUIRE guidelines and Declaration of Helsinki. This study was conducted with institutional approval from the Hokkaido University Hospital (No. 018-0291). As our facility regularly holds off-the-job microvascular anastomosis training sessions for educational purposes, surgeon participants, including both instructors and trainees, were recruited from these sessions. Fourteen surgeons with varying levels of microsurgical experience, ranging from postgraduate years (PGY) 1 to 28, participated in the experimental surgical performance analysis. Table 1 summarizes the characteristics of the participating surgeons. All participants were right-handed.

**Table 1:** Characteristics of participating surgeons.

Surgeon No	PGY	Experience (N)			Total points in criteria-based scale scores
		Micro-neurosurgery	EC-IC bypass	Laboratory training	
1	28	> 2000	> 300	> 50	42.0
2	20	> 1500	> 150	> 50	43.7
3	14	> 250	> 20	> 30	41.7
4	13	> 900	> 90	> 30	43.3
5	12	> 600	> 80	> 30	42.3
6	8	< 200	< 10	> 30	39.0
7	6	< 100	< 5	< 20	30.3
8	3	< 30	0	< 20	26.0
9	3	< 40	0	< 15	24.3
10	2	< 30	0	< 5	24.7
11	2	< 20	0	< 5	22.7
12	2	< 10	0	< 5	17.7
13	1	< 5	0	< 2	29.3
14	1	< 5	0	< 10	24.0

All participants were right-handed. The experience column indicates the number of surgical cases performed or laboratory microsurgery training courses (over 2 h) attended by each participant.

*N* number, *PGY* postgraduate year, *EC-IC* extracranial-intracranial.

### Microsurgical task

**Fig. 2:**
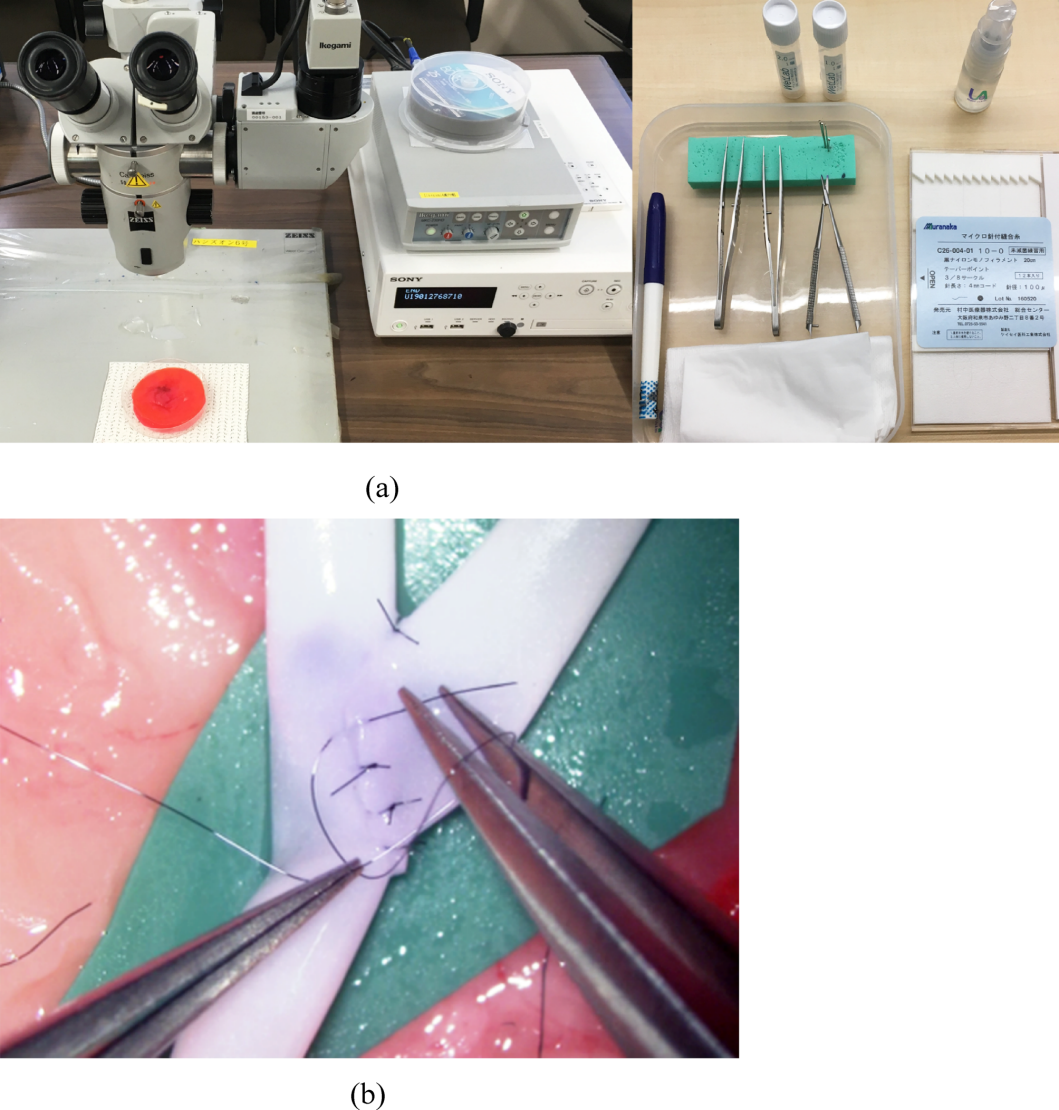
(**a**) Microscope, video recording device, and instruments. (**b**) Single-stitch suturing task for end-to-side anastomosis.

Each surgeon was assigned to perform interrupted suturing following two stay sutures in end-to-side anastomosis using artificial blood vessels (2.0 mm blood vessel model, WetLab Incorporated, Shiga, Japan) and 10-0 nylon monofilament threads (C26-004-01, Muranaka Medical Instruments Co., Ltd, Osaka, Japan), which assimilated the actual EC-IC bypass procedure (Fig. 2). To standardize the anastomosis procedure and minimize procedural variability, tasks such as stabilizing the two vessels, cutting the donor artery, performing arteriotomy on the recipient vessel, and preparing two stay sutures were performed and confirmed by a single instructor. A surgical trial was defined as the completion of a single suturing process, consisting of four phases: Phase A, grasping and inserting the needle; Phase B, pushing and extracting the needle; Phase C, pulling the threads to the first knot; and Phase D, tying three knots and cutting the threads^25^. To minimize the learning effect of repeated trials, only the first two trials from each surgeon were selected for the performance assessment.

### Criteria-based objective assessment

The Stanford Microsurgery and Resident Training Scale was used to assess each surgeon’s performance^10,11^. This rating scale consists of nine technical categories: (1) instrument handling, (2) respect for tissue, (3) efficiency, (4) suture handling, (5) suturing technique, (6) quality of the knot, (7) final product, (8) operation flow, and (9) overall performance. Three experts independently rated all surgeons’ performances on a scale of 1 to 5 for each technical category in a blinded manner, ensuring that the participants’ identities remained concealed. The score from the first two trials averaged across the three raters was used as the representative score for each surgeon.

### Video parameters of dual AI model

Table 2 presents the parameters generated by the combined AI model. These include the coefficient of variation (CV) of all measured VA values (CV-VA), the relative change in VA over time (ΔVA), the maximum absolute value of ΔVA during the procedure (Max-ΔVA), the number of tissue deformation errors (No. of TDE), the path distance (PD) for the right and left forceps tips, and the normalized jerk index (NJI) for the right and left forceps tips^24,25^. As the mean ± 1.96 × SD of ΔVA for all trials by all surgeons was calculated as ± 1.13 in a previous study, this threshold was used for the definition of TDE^24^.

**Table 2:** Definitions of parameters provided by the artificial intelligence (AI) model.

Algorithm	Abbreviation	Definition/equation
Semantic segmentation algorithm for vessel area (VA)	CV-VA	The coefficient of variation (CV) of all measured VA values VA = Total number of pixels in the vessel area CV-VA = σ/μ σ: standard deviation (SD) of VA, μ: mean value of VA
	ΔVA	The relative change in VA over time ΔVA = dVA/dt t: time
	Max-ΔVA	Maximum change in vessel area: The highest absolute value of ΔVA during the procedure
	TDE	Tissue deformation error: Any ΔVA values that exceed the defined threshold Threshold: Mean ± 1.96 × SD of ΔVA, calculated across all trials by all surgeons (± 1.13)
Trajectory tracking algorithm for instrument-tip	PD	Path distance: The total distance traveled by the forceps tip during the procedure
	NJI	Normalized jerk index: A measure of motion smoothness, calculated based on acceleration and procedural time ${\text{NJI}} = \frac{1}{2}*\frac{{t^{5} }}{{D^{2} }}*\smallint \left( {\frac{da}{{dt}}} \right)^{2}$ a: acceleration, t: procedural time, D: path distance

We analyzed videos from all surgeons’ trials and calculated each parameter. The average of the first two trials was used as the representative parameter for each surgeon.

### Statistical analysis

The results are expressed as the mean ± standard deviation. The interrater reliability of the criteria-based objective rating scale was assessed using Cronbach’s α coefficient among the three raters. Correlations between the AI-based parameters and each category of the rating scale were analyzed using Spearman’s rank correlation coefficient (ρ).

To assess the discriminative ability of each AI model’s parameters, we conducted discriminant analyses between the *good-* and *poor-performance* groups stratified by the distribution of the criteria-based rating scale scores of the surgeons. For each AI model, we selected the parameter that showed a significant correlation (*p* < 0.05) with the highest number of technical categories and included it in discriminant analysis (Models 1 and 2). Finally, to evaluate the combined use of both AI models, all the parameters from Models 1 and 2 were included in the discriminant analysis (Model 3).

Statistical analyses were performed using JMP Pro (version 17.0.0; SAS Institute Inc., Cary, North Carolina, USA). Statistical significance was set at *p* < 0.05.

## Results

### Criteria-based scale

The high interrater reliability for the criteria-based rating scale was confirmed with a Cronbach’s α coefficient of 0.88–0.97 for each category (Supplementary Table 1). The original criteria-based scale scores for each surgeon are provided in Table 1, and detailed in Supplementary Table 2.

**Fig. 3:**
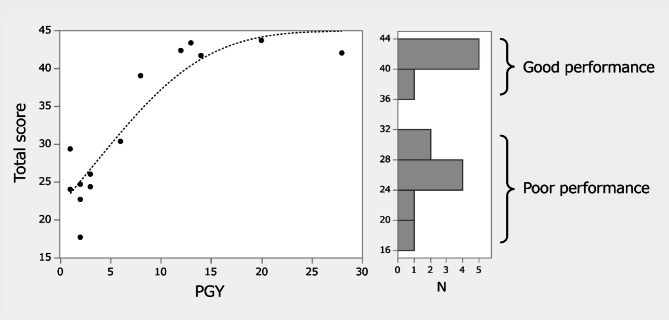
Distribution of criteria-based rating scale scores across surgeons with varying experience levels, along with the criteria used to define good and poor performance. *PGY* postgraduate year, *N* number.

The total score of the criteria-based rating scale significantly correlated with the surgeon’s experience (Fig. 3). As the total score on the criteria-based rating scale across the 14 surgeons exhibited a bimodal distribution, performances that scored over 35 points were regarded as *good*, whereas trials that scored under 35 were regarded as *poor* (Fig. 3).

### Correlation between AI-based and criteria-based performance analysis

Supplementary Table 3 provides the AI-derived performance parameters for each surgeon. Table 3 presents the *p*-values from the Spearman’s rank correlation analyses, and Supplementary Table 4 provides the Spearman’s rank correlation coefficient (ρ).

**Table 3:** Spearman’s rank correlation analysis (*p*-values) between the criteria-based scale and parameters provided by the AI model, including selected parameters for discriminant analysis.

Parameter	Phase	IH	RT	Ef	SH	ST	QK	FP	OF	OP	M1	M2	M3
No. of TDE	All	0.01*	0.02*	0.04*	0.13	0.07	0.27	0.09	0.09	0.02*			
	A	0.05	0.10	0.30	0.36	0.15	0.14	0.12	0.31	0.09			
	B	0.12	0.14	0.35	0.52	0.34	0.34	0.29	0.42	0.17			
	C	< 0.01**	0.03*	0.05*	0.10	0.04*	0.18	0.04*	0.06	< 0.01**	In		In
	D	0.02*	0.11	0.12	0.26	0.11	0.33	0.10	0.18	0.07			
CV-VA	All	0.26	0.19	0.38	0.39	0.38	0.99	0.59	0.64	0.39			
	A	0.34	0.41	0.68	0.73	0.52	0.96	0.68	0.95	0.76			
	B	0.09	0.06	0.16	0.25	0.18	0.50	0.22	0.26	0.17			
	C	0.22	0.21	0.32	0.39	0.34	1.00	0.59	0.57	0.25			
	D	0.08	0.09	0.16	0.29	0.20	0.61	0.24	0.24	0.11			
Max-ΔVA	All	0.07	0.19	0.22	0.14	0.12	0.47	0.26	0.39	0.24			
	A	0.05	0.28	0.45	0.28	0.08	0.18	0.10	0.33	0.17			
	B	0.03*	0.04*	0.11	0.19	0.08	0.11	0.08	0.14	0.07	In		In
	C	0.34	0.52	0.69	0.54	0.42	0.89	0.71	0.89	0.63			
	D	0.07	0.18	0.08	0.16	0.13	0.52	0.14	0.16	0.16			
Rt-PD	All	0.02*	0.01*	< 0.01**	< 0.01**	< 0.01**	0.09	0.02*	< 0.01**	< 0.01**		In	In
	A	0.82	0.61	0.64	0.54	0.58	0.82	0.81	0.52	0.48			
	B	0.12	0.02*	0.06	0.10	0.17	0.39	0.29	0.09	0.14			
	C	0.07	0.08	0.12	0.09	0.04*	0.15	0.03*	0.01*	0.04*			
	D	0.05	0.04*	0.02*	0.05*	0.09	0.24	0.11	0.02*	0.06			
Rt-NJI	All	0.18	0.10	0.05	0.04*	0.05	0.02*	0.05*	0.02*	0.02*			
	A	0.74	0.83	0.78	0.86	0.89	0.64	0.71	0.61	0.74			
	B	0.76	0.22	0.34	0.26	0.66	0.31	0.70	0.42	0.36			
	C	0.07	< 0.01**	0.01*	0.06	0.04*	0.05*	0.02*	< 0.01**	0.01*		In	In
	D	0.02*	0.13	0.03*	0.04*	0.02*	< 0.01**	< 0.01**	0.06	0.01*			
Lt-PD	All	0.10	0.15	0.04*	0.02*	0.02*	0.19	0.07	0.04*	0.08		In	In
	A	0.94	0.97	0.49	0.64	0.72	0.96	0.76	0.61	0.67			
	B	0.66	0.33	0.43	0.44	0.71	0.63	0.97	0.47	0.28			
	C	0.16	0.19	0.20	0.09	0.05*	0.15	0.06	0.03*	0.10			
	D	0.22	0.42	0.20	0.14	0.22	0.63	0.40	0.48	0.50			
Lt-NJI	All	0.02*	< 0.01**	< 0.01**	< 0.01**	< 0.01**	< 0.01**	< 0.01**	< 0.01**	< 0.01**		In	In
	A	0.39	0.05*	0.28	0.21	0.26	0.23	0.31	0.07	0.16			
	B	0.64	0.05*	0.14	0.12	0.47	0.43	0.57	0.16	0.34			
	C	0.49	0.11	0.24	0.54	0.62	0.31	0.38	0.22	0.30			
	D	0.05	0.07	0.05	0.08	0.07	0.01*	0.02*	0.02*	< 0.01**			

*IH* instrument handling, *RT* respect for tissue, *Ef.* efficiency, *SH* suture handling, *ST* suturing technique, *QK* quality of knot, *FP* final product, *OF* operation flow, *OP* overall performance, *M* model, *p < 0.05, **p < 0.01.

The No. of TDE for all phases and Phase C were significantly correlated with instrument handling, respect for tissue, efficiency and overall performance. In addition, the No. of TDE for Phase C was significantly correlated with suturing technique and final product. Although CV-VA and Max-ΔVA for all phases did not show significant correlations with technical categories, Max-ΔVA for Phase B was significantly correlated with instrument handling and respect for tissue. Therefore, the No. of TDE for Phase C and Max-ΔVA for Phase B were included in Models 1 and 3 for subsequent discriminant analysis.

The PD of the right forceps (Rt-PD) and the NJI of the left forceps (Lt-NJI) for all phases were significantly correlated with almost all performance categories. The NJI of the right forceps (Rt-NJI) for all phases was significantly correlated with five technical categories, while the Rt-NJI for Phase C and Phase D were significantly correlated with seven categories. The PD of the left forceps (Lt-PD) was significantly correlated with efficiency, suture handling, suturing technique, and operation flow. Therefore, Rt-PD, Lt-PD, and Lt-NJI for all phases and Rt-NJI for Phase C were included in Models 2 and 3 for the subsequent discriminant analysis.

### Discriminative abilities of each model for surgical performance

Supplementary Table 5 provides the discriminant functions of Models 1–3.

**Fig. 4:**
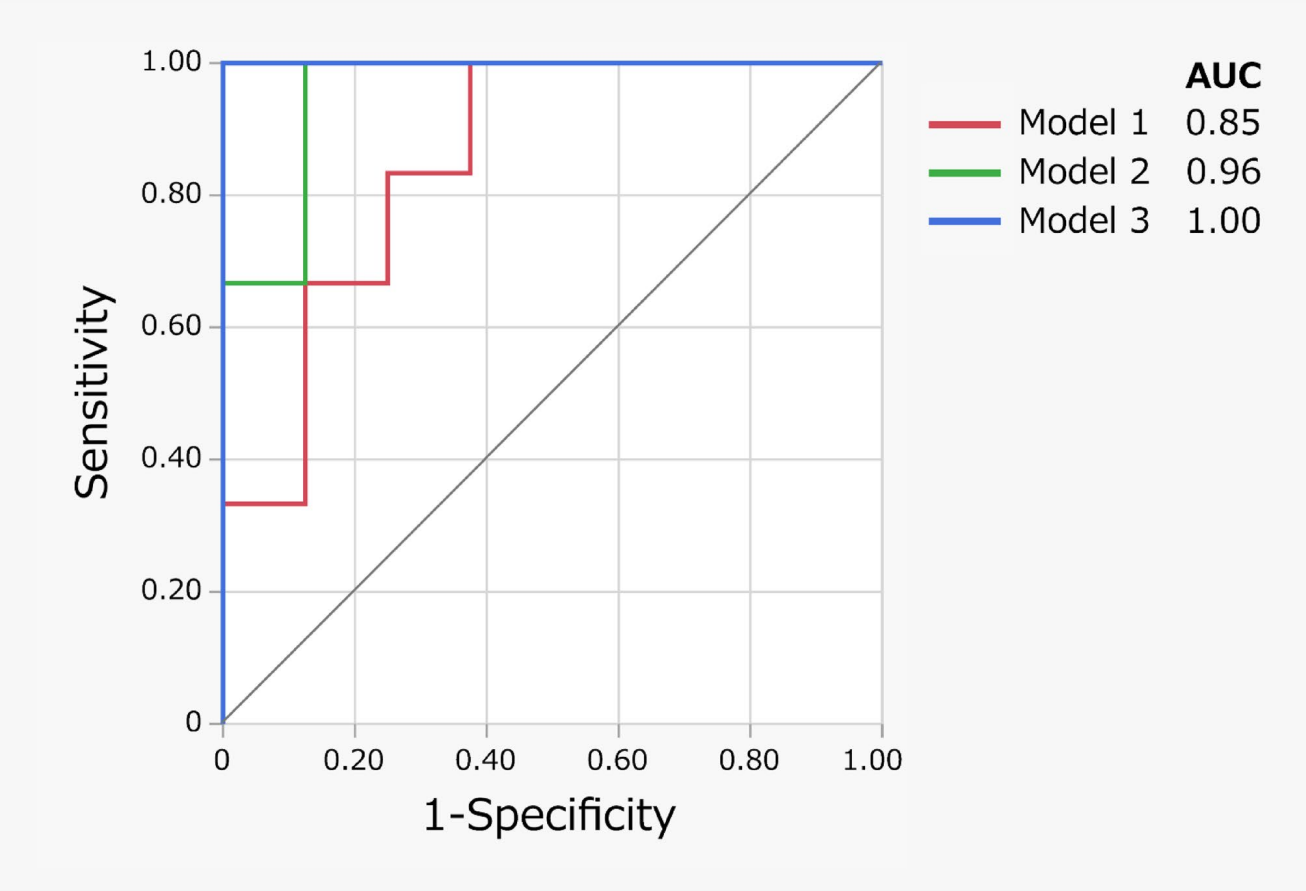
Receiver operating characteristic curves for Models 1–3, illustrating their ability to differentiate between good and poor performance. *AUC* area under the curve.

The receiver operating characteristic (ROC) curves for Models 1–3 used to distinguish between *good* and *poor performance* are shown in Fig. 4. The AUC values for Models 1 and 2 were 0.85 (95% confidence interval [CI]: 0.53–0.97) and 0.96 (95% CI: 0.67–1.00), respectively. Model 3 demonstrated the highest AUC value of 1.00.

## Discussion

We employed a combined AI-based video analysis approach to assess the microvascular anastomosis performance by integrating VA changes and instrument motion. By comparing technical category scores with AI-generated parameters, we demonstrated that the parameters from both AI models encompassed a wide range of technical skills required for microvascular anastomosis. Furthermore, ROC curve analysis indicated that integrating parameters from both AI models improved the ability to distinguish surgical performance compared to using a single AI model. A distinctive feature of this study was the integration of multiple AI models that incorporated both tools and tissue elements.

### AI-based technical analytic approach

Traditional criteria-based scoring by multiple blinded expert surgeons was a highly reliable method for assessing surgeon performance with minimal interrater bias (Fig. 2 and Supplementary Table 1). However, the significant demand for human expertise and time makes real-time feedback impractical during surgery and training^10,11,18^. A recent study demonstrated that self-directed learning using digital instructional materials provides non-inferior outcomes in the initial stages of microsurgical skill acquisition compared to traditional instructor-led training^28^. However, direct feedback from an instructor continues to play a critical role when progressing toward more advanced skill levels and actual clinical practice.

AI technology can rapidly analyze vast amounts of clinical data generated in modern operating theaters, offering real-time feedback capabilities. The proposed method’s reliance on surgical video analysis makes it highly applicable in clinical settings^18^. Moreover, the manner in which AI is utilized in this study addresses concerns regarding transparency, explainability, and interpretability, which are fundamental risks associated with AI adoption. One anticipated application is AI-assisted devices that can promptly provide feedback on technical challenges, allowing trainees to refine their surgical skills more effectively^29,30^. Additionally, an objective assessment of microsurgical skills could facilitate surgeon certification and credentialing processes within the medical community.

Theoretically, this approach could help implement a real-time warning system, alerting surgeons or other staff when instrument motion or tissue deformation exceeds a predefined safety threshold, thereby enhancing patient safety^17,31^. However, a large dataset of clinical cases involving adverse events such as vascular injury, bypass occlusion, and ischemic stroke would be required. For real-time clinical applications, further data collection and computational optimization are necessary to reduce processing latency and enhance practical usability. Given that our AI model can be applied to clinical surgical videos, future research could explore its utility in this context.

### Related works: AI-integrated instrument tracking

To contextualize our results, we compared our AI-integrated approach with recent methods implementing instrument tracking in microsurgical practice. Franco-González et al. compared stereoscopic marker-based tracking with a YOLOv8-based deep learning method, reporting high accuracy and real-time capability^32^. Similarly, Magro et al. proposed a robust dual-instrument Kalman-based tracker, effectively mitigating tracking errors due to occlusion or motion blur^33^. Koskinen et al. utilized YOLOv5 for real-time tracking of microsurgical instruments, demonstrating its effectiveness in monitoring instrument kinematics and eye-hand coordination^34^.

Our integrated AI model employs semantic segmentation (ResNet-50) for vessel deformation analysis and a trajectory-tracking algorithm (YOLOv2) for assessment of instrument motion. The major advantage of our approach is its comprehensive and simultaneous evaluation of tissue deformation and instrument handling smoothness, enabling robust and objective skill assessment even under challenging conditions, such as variable illumination and partial occlusion. YOLO was selected due to its computational speed and precision in real-time object detection, making it particularly suitable for live microsurgical video analysis. ResNet was chosen for its effectiveness in detailed image segmentation, facilitating accurate quantification of tissue deformation. However, unlike three-dimensional (3D) tracking methods^32^, our current method relies solely on 2D imaging, potentially limiting depth perception accuracy.

These comparisons highlight both the strengths and limitations of our approach, emphasizing the necessity of future studies incorporating 3D tracking technologies and expanded datasets to further validate and refine AI-driven microsurgical skill assessment methodologies.

### Future challenges

Microvascular anastomosis tasks typically consist of distinct phases, including vessel preparation, needle insertion, suture placement, thread pulling, and knot tying. As demonstrated by our video parameters for each surgical phase (phases A–D), a separate analysis of each surgical phase is essential to enhance skill evaluation and training efficiency. However, our current AI model does not have the capability to automatically distinguish these surgical phases.

Previous studies utilizing convolutional neural networks (CNN) and recurrent neural networks (RNN) have demonstrated high accuracy in recognizing surgical phases and steps, particularly through the analysis of intraoperative video data^35,36^. Khan et al. successfully applied a combined CNN-RNN model to achieve accurate automated recognition of surgical workflows during endoscopic pituitary surgery, despite significant variability in surgical procedures and video appearances^35^. Similarly, automated operative phase and step recognition in vestibular schwannoma surgery further highlights the ability of these models to handle complex and lengthy surgical tasks^36^. Such methods could be integrated into our current AI framework to segment and individually evaluate each distinct phase of microvascular anastomosis, enabling detailed performance analytics and precise feedback.

Furthermore, establishing global standards for video recording is critical for broadly implementing and enhancing computer vision techniques in surgical settings. Developing guidelines for video recording that standardize resolution, frame rate, camera angle, illumination, and surgical field coverage can significantly reduce algorithmic misclassification issues caused by shadows or instrument occlusion^18,37^. Such standardization ensures consistent data quality, crucial for training accurate and widely applicable AI models across diverse clinical settings ^37^. These guidelines would facilitate large-scale data sharing and collaboration, substantially improving the reliability and effectiveness of AI-based surgical assessment tools globally.

### Technical consideration

The semantic segmentation AI models were designed to assess respect for tissue during the needle manipulation process^24^. As expected, the Max-ΔVA correlated with respect for tissue in Phase B (from needle insertion to extraction). Proper needle extraction requires following its natural curve to avoid tearing the vessel wall^6,7^, and these technical nuances were well captured by these parameters. Additionally, the No. of TDE correlated with respect for tissue in Phases C, indicating that even during the process of pulling the threads, surgeons must exercise caution to prevent thread-induced vessel wall injury^6,7^. These parameters also correlated with instrument handling, efficiency, suturing technique and overall performance—an expected finding, as proper instrument handling and suturing technique are fundamental to respecting tissue. Thus, the technical categories are interrelated and mutually influential.

Trajectory-tracking AI models were designed to assess motion economy and the smoothness of surgical instrument movements^25^. Motion economy can be represented by the PD during a procedure. The smoothness and coordination of movement are frequently assessed using jerk-based metrics, where jerk is defined as the time derivative of acceleration. Since these jerk indexes are influenced by both movement duration and amplitude, we utilized the NJI, first proposed by Flash and Hogan^38^. The NJI is calculated by multiplying the jerk index by [(duration interval)^5^/(path length)^2^], with lower values indicating smoother movements. The dimensionless NJI has been used as a quantitative metric to evaluate movement irregularities in various contexts, such as jaw movements during chewing^39,40^, laparoscopic skills^41^, and microsurgical skills^16,25^. In this study, the Rt-PD and Lt-NJI correlated with a broad range of technical categories. Despite their distinct roles in microvascular anastomosis, coordinated bimanual manipulation is essential for optimal surgical performance^6,7^. With regard to Rt-NJI, these trends were particularly evident in Phases C and D, highlighting the importance of the motion smoothness in thread pulling and tying knots in determining overall surgical proficiency.

Overall, integrating these parameters enabled a comprehensive assessment of complex microsurgical skills, as each parameter captured different technical aspects. Despite its effectiveness, the model still exhibited some degree of misclassification when differentiating between *good* and *poor performance*. Notably, procedural time—a key determinant of surgical performance^24,25^—was intentionally excluded from the analysis. Although further exploration of additional parameters remains essential, integrating procedural time could significantly improve the classification accuracy.

This study employed the Stanford Microsurgery and Resident Training scale^10,11^ as a criteria-based objective assessment tool, as it covers a wide range of microsurgical technical aspects. Future research incorporating leakage tests or the Anastomosis Lapse Index^13^, which identifies ten distinct types of anastomotic errors, could provide deeper insights into the relationship between the quality of the final product and various technical factors.

### Limitations

As mentioned above, a fundamental technical limitation of this analytical approach is the lack of 3D kinematic data, particularly in the absence of depth information. Another constraint was that when the surgical tool was outside the microscope’s visual field, kinematic data of the surgical instrument could not be captured^25^. Additionally, the semantic segmentation model occasionally misclassified images containing shadows from surgical instruments or hands^24^. To mitigate this issue, future studies should expand the training dataset to include shadowed images, thereby improving model robustness. Given that the AI model in this study utilized the ResNet-50 and YOLOv2 networks, further investigation is warranted to optimize network architecture selection. Exploring alternative deep learning models or fine-tuning existing architectures could further improve the accuracy and generalizability of surgical video analysis^18^.

Our study had a relatively small sample size with respect to the number of participating surgeons, although it included surgeons with a diverse range of skills. Moreover, we did not evaluate the data from repeated training sessions to estimate the learning curve or determine whether feedback could enhance training efficacy. Future studies should evaluate the impact of AI-assisted feedback on the learning curve of surgical trainees and assess whether real-time performance tracking leads to more efficient skill acquisition.

## Conclusion

A combined AI-based video analysis approach incorporating VA changes and instrument motion effectively captured a broad spectrum of microsurgical technical skills and evaluated microvascular anastomosis performance. Moreover, this approach is highly adaptable to clinical applications, can advance computer-assisted surgical education, and contributes to the improvement of patient safety.

## Supplementary Information

Below is the link to the electronic supplementary material.

Supplementary Material 1

## Data Availability

Due to privacy and ethical restrictions, the dataset is not publicly available. The data and materials supporting the findings of this study are available from the corresponding author upon reasonable request.
