# Supplementary material for: Artificial intelligence-integrated video analysis of vessel area changes and instrument motion for microsurgical skill assessment
Source: Sci Rep. 2025 Jul 31;15:27898. doi: 10.1038/s41598-025-13522-1 (PMC12314103; doi:10.1038/s41598-025-13522-1)
Supplement: Supplementary file 1 — Supplementary Material 1 [file 41598_2025_13522_MOESM1_ESM.docx]

|  | **Cronbach's α** |
| --- | --- |
| **Instrument handling** | 0.92 |
| **Respect for tissue** | 0.90 |
| **Efficiency** | 0.94 |
| **Suture handling** | 0.88 |
| **Suturing technique** | 0.94 |
| **Quality of knot** | 0.91 |
| **Final Product** | 0.94 |
| **Operation flow** | 0.92 |
| **Overall performance** | 0.92 |
| **Total points** | 0.97 |

**Supplementary Table 1** Inter-rater reliability

| **Surgeon No.** | 1 | 2 | 3 | 4 | 5 | 6 | 7 | 8 | 9 | 10 | 11 | 12 | 13 | 14 |
| --- | --- | --- | --- | --- | --- | --- | --- | --- | --- | --- | --- | --- | --- | --- |
| **PGY** | 28 | 20 | 14 | 13 | 12 | 8 | 6 | 3 | 3 | 2 | 2 | 2 | 1 | 1 |
| **Instrument handling** | 5.0 | 5.0 | 4.3 | 4.7 | 4.7 | 4.3 | 3.3 | 3.0 | 2.3 | 2.7 | 2.7 | 2.3 | 3.0 | 2.7 |
| **Respect for tissue** | 4.3 | 5.0 | 4.0 | 5.0 | 5.0 | 4.7 | 2.7 | 2.3 | 2.7 | 3.3 | 2.7 | 1.3 | 3.0 | 3.7 |
| **Efficiency** | 4.0 | 5.0 | 4.7 | 4.7 | 4.0 | 4.0 | 3.0 | 2.3 | 2.3 | 2.7 | 2.7 | 2.0 | 3.3 | 2.7 |
| **Suture handling** | 5.0 | 5.0 | 5.0 | 5.0 | 4.0 | 4.3 | 3.7 | 2.3 | 3.0 | 2.3 | 2.7 | 2.0 | 3.3 | 3.0 |
| **Suturing technique** | 5.0 | 5.0 | 4.7 | 4.7 | 4.7 | 4.0 | 3.7 | 2.7 | 2.7 | 2.3 | 2.3 | 1.7 | 3.3 | 2.3 |
| **Quality of knot** | 5.0 | 4.0 | 5.0 | 4.7 | 5.0 | 4.7 | 3.3 | 4.0 | 2.7 | 2.3 | 2.0 | 2.3 | 3.3 | 2.7 |
| **Final Product** | 5.0 | 5.0 | 5.0 | 4.7 | 5.0 | 4.3 | 4.0 | 4.0 | 3.0 | 3.0 | 2.3 | 2.3 | 3.3 | 2.3 |
| **Operation flow** | 4.0 | 5.0 | 4.7 | 5.0 | 5.0 | 4.3 | 3.3 | 2.7 | 3.0 | 3.0 | 2.3 | 1.7 | 3.3 | 2.7 |
| **Overall performance** | 4.7 | 4.7 | 4.3 | 5.0 | 5.0 | 4.3 | 3.3 | 2.7 | 2.7 | 3.0 | 3.0 | 2.0 | 3.3 | 2.0 |
| **Total points** | 42.0 | 43.7 | 41.7 | 43.3 | 42.3 | 39.0 | 30.3 | 26.0 | 24.3 | 24.7 | 22.7 | 17.7 | 29.3 | 24.0 |

**Supplementary Table 2** The original criteria-based scale scores assessing each surgeon’s microsurgical performance.

PGY, postgraduate year

| **Surgeon No.** | | 1 | 2 | 3 | 4 | 5 | 6 | 7 | 8 | 9 | 10 | 11 | 12 | 13 | 14 |
| --- | --- | --- | --- | --- | --- | --- | --- | --- | --- | --- | --- | --- | --- | --- | --- |
| **Parameter** | **Phase** |  |  |  |  |  |  |  |  |  |  |  |  |  |  |
| No. of TDE | All | 42.0 | 29.0 | 206.0 | 34.5 | 38.5 | 115.5 | 228.5 | 57.5 | 480.0 | 84.5 | 37.5 | 609.0 | 467.5 | 153.0 |
|  | A | 1.0 | 6.0 | 29.5 | 4.5 | 1.5 | 21.0 | 79.5 | 2.0 | 34.0 | 9.0 | 3.0 | 218.0 | 71.0 | 21.0 |
|  | B | 14.0 | 10.0 | 51.0 | 9.0 | 3.0 | 21.0 | 98.5 | 4.0 | 130.0 | 31.0 | 3.0 | 134.0 | 177.0 | 20.5 |
|  | C | 9.5 | 7.5 | 49.0 | 12.0 | 7.0 | 34.5 | 16.0 | 37.0 | 105.5 | 26.0 | 11.0 | 82.0 | 115.0 | 54.0 |
|  | D | 17.5 | 5.5 | 76.5 | 9.0 | 27.0 | 39.0 | 34.5 | 14.5 | 210.5 | 18.5 | 20.5 | 175.0 | 104.5 | 57.5 |
| CV-VA [/100] | All | 2.38 | 2.43 | 4.26 | 3.85 | 3.52 | 5.77 | 4.01 | 4.44 | 4.21 | 3.30 | 2.57 | 5.27 | 7.39 | 2.94 |
|  | A | 2.21 | 2.12 | 4.64 | 3.03 | 2.94 | 5.65 | 3.50 | 2.77 | 3.04 | 2.78 | 2.38 | 4.67 | 4.66 | 2.32 |
|  | B | 2.06 | 1.52 | 3.96 | 3.75 | 1.86 | 5.88 | 4.00 | 4.19 | 4.54 | 3.30 | 2.95 | 5.78 | 5.83 | 2.40 |
|  | C | 1.75 | 1.98 | 4.10 | 3.27 | 2.20 | 5.39 | 3.21 | 4.50 | 3.94 | 2.96 | 1.95 | 5.11 | 3.60 | 2.66 |
|  | D | 2.26 | 1.98 | 3.95 | 1.97 | 2.97 | 5.19 | 3.63 | 2.86 | 4.05 | 2.18 | 2.38 | 5.70 | 5.49 | 3.09 |
| Max-ΔVA | All | 2.94 | 2.15 | 4.74 | 3.19 | 4.37 | 4.87 | 4.27 | 3.74 | 4.44 | 4.78 | 3.36 | 5.71 | 5.74 | 3.38 |
|  | A | 1.09 | 2.14 | 3.59 | 2.21 | 2.15 | 3.10 | 2.70 | 1.62 | 2.70 | 3.24 | 2.50 | 4.34 | 4.60 | 2.92 |
|  | B | 2.61 | 1.83 | 2.91 | 2.40 | 1.25 | 2.82 | 4.15 | 2.01 | 3.20 | 3.62 | 2.20 | 4.34 | 3.17 | 2.66 |
|  | C | 2.22 | 1.66 | 4.27 | 2.52 | 3.78 | 3.77 | 3.74 | 2.75 | 2.89 | 3.17 | 2.33 | 3.95 | 4.81 | 3.06 |
|  | D | 2.94 | 1.63 | 3.02 | 2.43 | 3.67 | 4.67 | 2.91 | 3.15 | 4.44 | 2.23 | 3.25 | 4.90 | 4.33 | 3.15 |
| Rt-PD (mm) | All | 404.6 | 112.5 | 393.5 | 214.8 | 257.6 | 245.3 | 170.9 | 627.6 | 372.2 | 514.4 | 539.1 | 1230.0 | 776.3 | 521.7 |
|  | A | 54.3 | 44.1 | 54.4 | 31.8 | 57.7 | 29.3 | 48.2 | 50.5 | 21.7 | 46.6 | 30.3 | 373.3 | 70.3 | 146.9 |
|  | B | 98.6 | 33.0 | 77.3 | 46.2 | 36.6 | 53.3 | 59.3 | 90.4 | 83.7 | 192.2 | 40.1 | 239.1 | 108.2 | 36.7 |
|  | C | 72.3 | 42.3 | 81.5 | 44.6 | 33.4 | 82.8 | 11.4 | 175.7 | 53.4 | 67.1 | 265.0 | 189.2 | 285.1 | 176.1 |
|  | D | 207.8 | 34.8 | 121.9 | 76.2 | 86.3 | 71.8 | 29.1 | 167.7 | 186.4 | 165.7 | 131.6 | 631.2 | 256.1 | 122.6 |
| Rt-NJI [x10e9] | All | 2085.9 | 5287.1 | 196.7 | 1402.1 | 247.2 | 903.3 | 3098.4 | 5301.8 | 1386.0 | 3699.9 | 2422.5 | 42931.8 | 1332.6 | 12325.2 |
|  | A | 190.5 | 6.5 | 0.3 | 4.2 | 0.8 | 1.6 | 48.9 | 1.7 | 0.4 | 2.7 | 0.4 | 2187.9 | 1.7 | 24.8 |
|  | B | 27.3 | 1298.3 | 0.6 | 6.6 | 1.9 | 7.8 | 112.7 | 523.4 | 10.7 | 191.3 | 2.4 | 419.1 | 35.7 | 5.8 |
|  | C | 163.9 | 10.5 | 6.4 | 114.8 | 3.6 | 58.3 | 2996.9 | 420.8 | 161.1 | 62.1 | 310.0 | 629.1 | 40.6 | 884.4 |
|  | D | 16.6 | 81.7 | 7.3 | 45.9 | 15.3 | 23.6 | 52.5 | 34.6 | 90.4 | 84.5 | 29.0 | 571.1 | 31.7 | 546.6 |
| Lt-PD (mm) | All | 1939.0 | 887.8 | 1668.4 | 2182.5 | 1674.6 | 1169.4 | 1206.2 | 2802.1 | 1576.2 | 2816.4 | 1863.9 | 4825.8 | 2009.9 | 2665.8 |
|  | A | 955.8 | 240.2 | 100.2 | 594.1 | 245.8 | 220.1 | 411.1 | 359.0 | 166.5 | 424.1 | 93.2 | 2485.6 | 159.5 | 811.5 |
|  | B | 482.0 | 552.0 | 476.4 | 352.3 | 173.5 | 179.9 | 434.6 | 510.9 | 458.2 | 911.2 | 162.5 | 1315.8 | 254.5 | 425.8 |
|  | C | 303.8 | 299.0 | 407.8 | 442.5 | 265.0 | 192.7 | 43.5 | 669.5 | 151.6 | 371.0 | 999.6 | 1064.4 | 442.6 | 997.2 |
|  | D | 559.6 | 250.6 | 483.2 | 738.5 | 580.7 | 572.9 | 191.5 | 710.4 | 671.4 | 979.4 | 321.8 | 1429.7 | 1012.1 | 398.5 |
| Lt-NJI [x10e9] | All | 80.3 | 100.3 | 11.5 | 8.9 | 14.3 | 57.5 | 117.2 | 187.5 | 98.0 | 97.1 | 269.4 | 1957.1 | 192.9 | 149.6 |
|  | A | 0.2 | 0.2 | 0.2 | 0.0 | 0.1 | 0.1 | 1.0 | 0.2 | 0.0 | 0.1 | 0.4 | 69.0 | 0.7 | 0.1 |
|  | B | 10.8 | 1.7 | 0.0 | 0.2 | 0.4 | 1.6 | 5.7 | 32.8 | 0.5 | 5.4 | 0.6 | 31.1 | 20.8 | 0.0 |
|  | C | 21.6 | 11.5 | 0.3 | 0.7 | 0.1 | 381.1 | 390.4 | 24.6 | 33.0 | 3.4 | 25.7 | 9.4 | 6.1 | 6.0 |
|  | D | 1.3 | 15.8 | 0.7 | 0.3 | 1.0 | 0.6 | 2.2 | 1.5 | 7.2 | 1.0 | 6.4 | 47.6 | 2.2 | 120.9 |
| Procedural time (s) | All | 98.8 | 74.8 | 47.5 | 71.0 | 53.1 | 85.2 | 100.3 | 95.6 | 87.3 | 137.5 | 105.1 | 258.7 | 121.7 | 126.8 |
|  | A | 25.8 | 11.2 | 8.0 | 13.7 | 8.7 | 9.3 | 26.4 | 12.2 | 6.3 | 17.2 | 7.0 | 90.5 | 13.2 | 23.8 |
|  | B | 25.9 | 32.2 | 9.0 | 20.2 | 11.2 | 21.0 | 33.9 | 22.9 | 20.6 | 53.2 | 11.8 | 48.4 | 37.5 | 10.3 |
|  | C | 24.5 | 12.2 | 12.5 | 18.3 | 12.2 | 32.5 | 17.0 | 33.5 | 20.6 | 25.6 | 54.8 | 49.5 | 34.5 | 52.1 |
|  | D | 22.6 | 19.2 | 18.0 | 18.8 | 21.0 | 22.4 | 23.1 | 27.0 | 39.8 | 41.5 | 31.4 | 70.1 | 36.5 | 40.6 |

**Supplementary Table 3** AI-derived quantitative data for each surgeon’s performance.

|  |  | IH | RT | Ef. | SH | ST | QK | FP | OF | OP |
| --- | --- | --- | --- | --- | --- | --- | --- | --- | --- | --- |
| Parameter | Phase |  |  |  |  |  |  |  |  |  |
| No. of TDE | All | -0.64 | -0.63 | -0.55 | -0.42 | -0.49 | -0.32 | -0.47 | -0.47 | -0.61 |
|  | A | -0.53 | -0.46 | -0.30 | -0.26 | -0.41 | -0.42 | -0.44 | -0.29 | -0.47 |
|  | B | -0.44 | -0.41 | -0.27 | -0.19 | -0.28 | -0.27 | -0.30 | -0.24 | -0.39 |
|  | C | -0.70 | -0.59 | -0.54 | -0.46 | -0.56 | -0.38 | -0.56 | -0.52 | -0.69 |
|  | D | -0.62 | -0.45 | -0.44 | -0.32 | -0.45 | -0.28 | -0.45 | -0.38 | -0.51 |
| CV-VA | All | -0.32 | -0.37 | -0.25 | -0.25 | -0.25 | 0.00 | -0.16 | -0.14 | -0.25 |
|  | A | -0.27 | -0.24 | -0.12 | -0.10 | -0.19 | 0.01 | -0.12 | -0.02 | -0.09 |
|  | B | -0.47 | -0.52 | -0.40 | -0.33 | -0.38 | -0.20 | -0.35 | -0.32 | -0.39 |
|  | C | -0.35 | -0.36 | -0.28 | -0.25 | -0.28 | 0.00 | -0.16 | -0.17 | -0.33 |
|  | D | -0.48 | -0.47 | -0.39 | -0.30 | -0.36 | -0.15 | -0.33 | -0.34 | -0.44 |
| Max-ΔVA | All | -0.50 | -0.37 | -0.35 | -0.41 | -0.44 | -0.21 | -0.32 | -0.25 | -0.34 |
|  | A | -0.53 | -0.31 | -0.22 | -0.31 | -0.49 | -0.38 | -0.45 | -0.28 | -0.39 |
|  | B | -0.58 | -0.54 | -0.44 | -0.37 | -0.48 | -0.45 | -0.48 | -0.42 | -0.50 |
|  | C | -0.27 | -0.19 | -0.12 | -0.18 | -0.23 | 0.04 | -0.11 | -0.04 | -0.14 |
|  | D | -0.50 | -0.38 | -0.48 | -0.39 | -0.43 | -0.19 | -0.41 | -0.40 | -0.40 |
| Rt-PD | All | -0.63 | -0.66 | -0.67 | -0.71 | -0.69 | -0.47 | -0.62 | -0.77 | -0.66 |
|  | A | -0.07 | -0.15 | -0.14 | -0.18 | -0.16 | 0.07 | -0.07 | -0.19 | -0.20 |
|  | B | -0.44 | -0.60 | -0.51 | -0.46 | -0.39 | -0.25 | -0.30 | -0.47 | -0.42 |
|  | C | -0.50 | -0.48 | -0.43 | -0.47 | -0.56 | -0.41 | -0.58 | -0.64 | -0.56 |
|  | D | -0.53 | -0.56 | -0.61 | -0.54 | -0.47 | -0.34 | -0.45 | -0.60 | -0.51 |
| Rt-NJI | All | -0.38 | -0.46 | -0.53 | -0.57 | -0.52 | -0.59 | -0.53 | -0.62 | -0.61 |
|  | A | 0.10 | -0.06 | -0.08 | -0.05 | -0.04 | -0.14 | -0.11 | -0.15 | -0.10 |
|  | B | -0.09 | -0.35 | -0.28 | -0.32 | -0.13 | -0.29 | -0.11 | -0.23 | -0.27 |
|  | C | -0.50 | -0.66 | -0.66 | -0.52 | -0.56 | -0.53 | -0.63 | -0.72 | -0.66 |
|  | D | -0.61 | -0.42 | -0.57 | -0.55 | -0.60 | -0.72 | -0.66 | -0.52 | -0.66 |
| Lt-PD | All | -0.46 | -0.41 | -0.55 | -0.62 | -0.60 | -0.37 | -0.50 | -0.56 | -0.48 |
|  | A | 0.02 | -0.01 | -0.20 | -0.14 | -0.11 | -0.02 | -0.09 | -0.15 | -0.12 |
|  | B | -0.13 | -0.28 | -0.23 | -0.23 | -0.11 | -0.14 | -0.01 | -0.21 | -0.31 |
|  | C | -0.39 | -0.37 | -0.36 | -0.47 | -0.54 | -0.41 | -0.51 | -0.57 | -0.46 |
|  | D | -0.35 | -0.23 | -0.36 | -0.42 | -0.35 | -0.14 | -0.25 | -0.20 | -0.19 |
| Lt-NJI | All | -0.62 | -0.77 | -0.69 | -0.72 | -0.68 | -0.74 | -0.72 | -0.81 | -0.74 |
|  | A | -0.25 | -0.64 | -0.31 | -0.36 | -0.32 | -0.35 | -0.30 | -0.49 | -0.40 |
|  | B | -0.14 | -0.53 | -0.42 | -0.43 | -0.21 | -0.23 | -0.17 | -0.40 | -0.27 |
|  | C | -0.20 | -0.45 | -0.33 | -0.18 | -0.15 | -0.29 | -0.25 | -0.35 | -0.30 |
|  | D | -0.53 | -0.50 | -0.53 | -0.49 | -0.50 | -0.65 | -0.60 | -0.61 | -0.67 |

**Supplementary Table 4** Spearman's rank correlation coefficient (ρ) between the criteria-based scale and parameters provided by the AI model

IH; instrument handling, RT; respect for tissue; Ef.; Efficiency, SH; suture handling, ST; suturing technique, QK; quality of knot, FP; final product, OF; operation flow, OP; overall performance

|  | No. of TDE  (Phase C) | Max-ΔVA  (Phase B) | Rt-PD  (All phases) | Rt-NJI  (Phase C) | Lt-PD  (All phases) | Lt-NJI  (All phases) |
| --- | --- | --- | --- | --- | --- | --- |
| Model 1 | 0.849 | 0.864 |  |  |  |  |
| Model 2 |  |  | 0.695 | 0.501 | 0.562 | 0.424 |
| Model 3 | 0.601 | 0.613 | 0.681 | 0.490 | 0.551 | 0.415 |

**Supplementary Table 5** Canonical functions in Models 1-3
